# Can Public Health Interventions Change Immediate and Long-Term Dietary Behaviours? Encouraging Evidence from a Pilot Study of the U.K. Change4Life Sugar Swaps Campaign

**DOI:** 10.3390/nu14010068

**Published:** 2021-12-24

**Authors:** Daniel J. Lamport, Szu-Yun Wu, Jenni Drever-Heaps, Orla Hugueniot, Daniel J. W. Jones, Orla B. Kennedy, Claire M. Williams, Laurie T. Butler

**Affiliations:** 1 School of Psychology and Clinical Language Science, University of Reading, Reading RG6 6AL, UK; jenni_dh@yahoo.co.uk (J.D.-H.); dan.jones@reading.ac.uk (D.J.W.J.); claire.williams@reading.ac.uk (C.M.W.); 2 Institute of Biomedical Science, Academia Sinica, Taipei 11529, Taiwan; Sheilawusy@gmail.com; 3 Public Health England, London NW9 5EQ, UK; orla.hugueniot@phe.gov.uk; 4 School of Chemistry Food and Pharmacy, University of Reading, Reading RG6 6AL, UK; o.b.kennedy@reading.ac.uk; 5 Faculty of Science and Engineering, Anglia Ruskin University, Chelmsford CM1 1SQ, UK; laurie.butler@aru.ac.uk

**Keywords:** change for life, sugar swaps, dietary intervention, public health, nutrition, sugar

## Abstract

The aim of this pilot study was to evaluate the effectiveness of the U.K. Change4Life Sugar Swaps campaign for improving nutritional intake in a small sample of families prior to the 2015 nationwide launch. A total of 49 participants from 14 families received information and materials during a two-week intervention period in November 2014 encouraging them to swap high sugar foods and drinks for low sugar alternatives. Daily dietary intake was reported with online food diaries over four stages, each two weeks in length: (i) baseline (no information provided), (ii) intervention when Sugar Swaps materials were accessible, (iii) immediate follow-up, and (iv) one year on from baseline. Data were analysed for sugar, glucose, fructose, sucrose, lactose, fat, saturated fat, carbohydrate, protein, salt, fibre, vitamin C, and energy. During the intervention, significant daily reductions of 32 g sugar, 11 g fat, and 236 kcal for each family member were observed, among others, and 61% of benefits achieved during the intervention period were maintained at immediate follow-up. Encouragingly, for children, reductions in sugar, sucrose, fat, saturated fat, carbohydrate, and energy were observed one year on. The Sugar Swaps Campaign is potentially an effective public health intervention for improving short- and long-term dietary behaviour for the whole family.

## 1. Introduction

Increased sugar intake globally has contributed to a higher prevalence of obesity, and increased incidence of health disorders associated with excess calorie intake such as type 2 diabetes cardiovascular disease (CVD) and dental problems. For example, comprehensive meta-analyses show positive associations between dietary sugar intake and risk of weight gain [1] and CVD [2] and, in the U.K., an estimated 79,000 new cases of type 2 diabetes will be attributable to sugar sweetened beverage consumption between 2015 and 2025 [3]. Recently, sugar consumption has been identified as an independent risk factor for COVID-19-related mortality [4,5] Concerningly, public health records indicate that more than a third of U.K. children are overweight or obese [6,7]. Data support the assertion that, at a population level, reducing sugar intake will reduce levels of obesity and obesity-related disease [8].

With this in mind, in 2009, Public Health England launched the ‘Change4Life’ campaign, which was designed to engage the public and raise awareness of the impact of behavioural changes on health outcomes. It has included a variety of interventions focused on increasing exercise and reducing intake of saturated fat, salt, and sugar. Previous similar interventions in other countries have shown encouraging findings. A complex simulation modelling of low fat/sugar substitutions in the French diet indicated positive dietary changes without loss of nutritional quality [9].

Here in the U.K., the Change4Life campaign has shown some success. For example, the 2014 Change4Life Smart Swap campaign was associated with increased purchasing of healthier foods over a three-week period relative to a control group not exposed to the materials [10]. However, dietary intake was not assessed. Following on from this, a campaign focused specifically on reducing sugar intake (U.K. Change4Life Sugar Swaps) was publicly launched in January 2015. U.K. families were provided with information (online and by post) encouraging them to swap high sugar foods and drinks for low sugar alternatives. These ‘sugar swaps’ were encouraged for all members of the family, with the materials and information predominantly designed to reduce sugar intake in children. Prior to the nationwide public launch of Change4Life Sugar Swaps, this research was conducted, with the aim of evaluating the campaign’s potential effectiveness. Specifically, we collected detailed and comprehensive food diary datasets over eight weeks in a small sample of families exposed to the Sugar Swaps materials. To see long-term population reductions in incidence of obesity, type 2 diabetes, and CVD, effective public health campaigns must be able to show sustainable changes to dietary and behavioural habits in the months and years after the introduction of the initial promotional material. Therefore, in the present study, we evaluated immediate and long-term changes in dietary intake. It is noteworthy that, following the completion of this research, a new iteration of the 2015 Sugar Swaps campaign was launched in 2016, known as the Sugar Smart Campaign. Interestingly, the 2016, Sugar Smart campaign was shown to reduce sugar intake by 2% in children, but these benefits were not sustained a year later [11].

The specific objectives of this research were (i) to explore any immediate changes to family diet (not just sugar) during the introduction of the intervention and (ii) whether these changes could be maintained post intervention up to one year later. An additional objective was to undertake an analysis of different members of the family where applicable (e.g., male parent, female parent, and children) to determine for whom the campaign materials were most effective.

## 2. Materials and Methods

### 2.1. Design

There were four stages:

(i) Baseline: two weeks immediately prior to beginning Sugar Swaps.
(ii) Intervention: two weeks while families participated in Sugar Swaps.
(iii) Immediate follow-up: two weeks during which no dietary advice was provided.
(iv) One-year follow-up: a two-week period, one year later, during the same calendar weeks as the intervention.

A separate control group was not recruited because of the anticipated difficulties in matching the control and experimental families on demographics and dietary habits. Furthermore, it would not have been possible to prevent participants in the control group from interacting with the campaign materials following the nationwide launch. The two-week baseline served as the control for each family. Data were collected for various outcome measures including macronutrients, vitamin C, salt, and energy intake (see Analysis). In summary, data collection occurred daily over an initial period of six weeks, followed by a further two weeks 12 months later (Thursday 22 October 2015 to Wednesday 4 November 2015). For the initial 6-week period, data collection commenced on Thursday 23 October 2014 and ended on Wednesday 3 December 2014 (inclusive).

### 2.2. The Intervention

At the end of the 2-week baseline period, but prior to commencement of the intervention, the families were provided by post with information packs and guidance on how to implement ‘swaps’ into their diets. These packs (47 pages in length) included information and guidance for adults and children on what sugar is, sources and types of sugar, health risks of overconsuming sugar, foods and drinks that are high in sugar and what can be swapped as a low sugar alternative, low sugar recipes, understanding colour-coded food labelling, general tips for healthy eating, and celebrity endorsements. Examples and information were provided for all the family, while the general focus of the materials was towards reducing sugar for children. Large sections of the pack were categorised into reducing sugar at five key timepoints: breakfast, lunch at school, after school snacks, sugary drink swaps, and eating healthily on weekends. These packs were designed and distributed by Public Health England Change4Life team and matched those made publicly available at the U.K. launch on 4 January 2015 (see https://www.nhs.uk/change4life/food-facts/sugar (accessed on 2 November 2021) for the currently available packs and information).

### 2.3. Participants

The sample consisted of 49 participants (26 adults and 23 children) from 14 families. Of these 14, there were six one-child families, seven two-child families, and one three-child family, giving an average of 1.64 children per family. Two families did not include data for a male parent. The average self-reported heights and weights per family member collected at one year follow-up are shown in Table 1. Age or any other demographic data were not collected. To maximise generalisability to the U.K. population, the only inclusion/exclusion criteria were that families must live in the United Kingdom and have at least one child under 18 years of age. The families were recruited after responding to a free advertisement provided by the University of Reading research team on the U.K. social online community ‘Netmums.com’, requesting families to take part in a Change4Life healthy eating trial. A total of 54 families responded to the advertisement, and 44 families agreed to participate in all stages of the study. Further, 14 families provided sufficient data for all stages on account of withdrawal (*n* = 15 families) and missing data for 3 days or more over a 2-week period (*n* = 15 families). A power calculation was not undertaken based on an absence of suitably comparable published data sets and the strategy was to recruit as many families as possible in the short timeframe available prior to the nationwide launch.

### 2.4. Procedure

Interested participants responded to the Netmums.com advert, with their details then passed to the University of Reading research team, who then contacted participants by phone and/or email to explain the study in full, check eligibility, and obtain consent. During the baseline period, immediate follow-up, and one-year follow-up, no dietary guidance or information was provided, thus participants were free-living. On a daily basis, participants recorded everything they had consumed (foods and drinks) categorised by meal type (breakfast, lunch, evening meal, and snacks) during each two-week period (baseline, intervention, immediate follow-up, and one-year follow-up). One parent entered the dietary data for each family member into an online food diary at the end of each day, or at the earliest opportunity the following day using an electronic link provided daily by email. This email link included specific, detailed instructions on how to complete the food diary (with examples) and a meal-structured food diary for completion ‘on-the-go’ to minimise reliance on memory. Participants were instructed to provide detail on weight/volume, any branding, and preparation including foods/drinks consumed outside of the home at restaurants/parties, among others. The online diary was categorised into four sections; breakfast, lunch, evening meal, and snacks, for each member of the family (mum, dad, and each child). Participants were also required to indicate how many ‘swaps’ had been achieved by each member of the family for each day during the intervention period. A ‘swap’ was defined as replacing a high sugar item with a low sugar alternative based on examples provided in the PHE intervention packs (see above). Daily reminders were sent to participants who had failed to complete the food diary. The online diaries could not be saved as work in progress owing to software limitations. Upon completion of the study, each family received £300 in supermarket vouchers. The research was approved by the University of Reading School of Psychology Research Ethics committee(DL-2014).

### 2.5. Analysis

Unless otherwise stated, the term family refers to two adults and 1.64 children, as previously described. Quantitative nutritional data were extracted from the food diaries using Dietplan7 [12] for the following: sugar (all mono and disaccharides), glucose, fructose, sucrose, lactose, fat, saturated fat, carbohydrate, protein, salt, fibre, vitamin C, and energy (using McCance and Widdowson’s The Composition of Foods 2014). The number of drink and food swaps during the intervention was self-reported, and portions of fruit and vegetables (80 g) were calculated from food diary data. When participants consumed food outside of the home that could not be weighed, portion sizes were estimated with the FSA Food Portion Sizes reference book (2002, 3rd ed.) and Food Atlas (https://webarchive.nationalarchives.gov.uk/ukgwa/20100408093610/http://www.food.gov.uk/multimedia/pdfs/publication/foodatlaspreschool0310.pdf, accessed on 2 November 2021). Nutritional data were analysed with SPSS (Version 23, SPSS Inc., Chicago, IL, USA) for each member of the family, with 4 × 4 repeated measures ANOVAs with Timepoint (baseline, intervention, immediate follow-up, and year follow-up) and Meal (breakfast, lunch, evening meal, and snacks) as independent variables. Where significant effects were observed, post hoc tests were performed with Bonferroni corrections for type 1 error. Within each family, prior to the ANOVA analysis, data for children were calculated as average per child for consistency across families. For each stage of the analysis (see Design), data were averaged over 14 days for daily consumption. Swaps data were analysed with a repeated measures ANOVA with Family Member (male, female, and child), Swap Type (food and drink), and Meal (breakfast, lunch, evening meal, and snacks) as independent variables. For all analyses, *p* < 0.05 was considered statistically significant (after Bonferroni corrections where relevant). For cases of missing data, data were averaged across the number of days for which data were available.

## 3. Results

### 3.1. Nutritional Outcomes

Table 2 outlines average consumption per family member at each stage of the intervention and indicates where there are significant differences to baseline. Table 2 shows significant benefits on 36 occasions relative to baseline, notably for sugar and fat for all members of the family. The majority of these benefits were seen during the intervention (*n* = 18), while 11 benefits remained immediately post-intervention and 7 benefits persisted one year later. Therefore, 61% (11/18) of the benefits achieved during the initial two weeks were maintained during the two-week follow-up, and 38% (7/18) were maintained one year later. These benefits are discussed in more detail by family member below.

Table 3 extends Table 2 by showing where changes occurred for each meal type, and the quantifiable nature of the change relative to baseline. Mean (range) number of days data per family for each stage was 13.86 (12–14) for baseline, 13.93 (13–14) for intervention, 14 (14) for immediate follow-up, and 14 (14) for one year follow-up.

#### 3.1.1. Children—Specific Dietary Benefits

**Figure 1:**
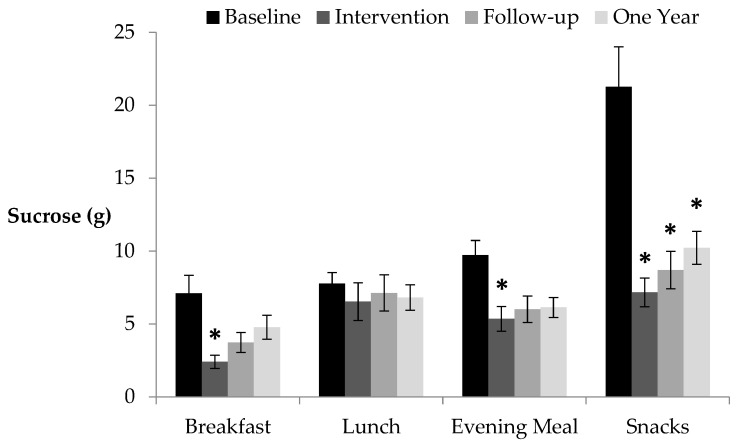
Sucrose consumption (g) average per child per day per meal time (se) at each two-week stage (* *p* < 0.05 Bonferroni corrected relative to baseline) following the Meal * Time interaction (F[9, 99] = 2.69, *p* < 0.01).

Children were clearly the family member most likely to benefit from this campaign, with notable reductions in sugars and fats (see Table 2 and Table 3). In particular, sucrose consumption was reduced at all time points relative to baseline and, as Figure 1 illustrates, a reduction in sucrose consumption from snacks accounted for the majority of these savings.

**Figure 2:**
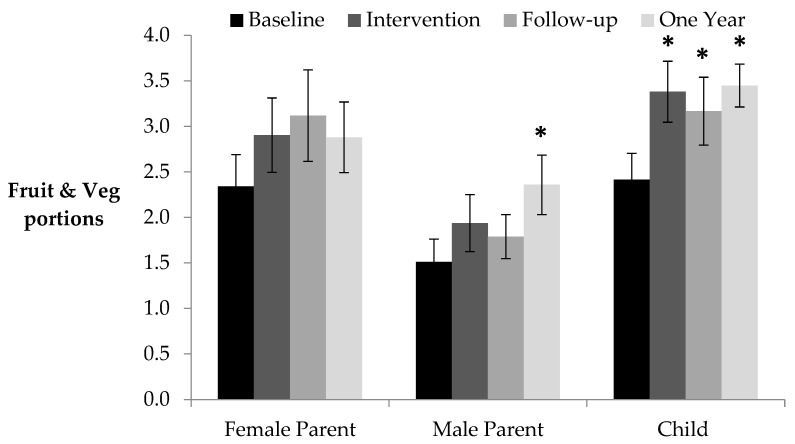
Fruit and vegetable consumption portions (80 g) per day (se) average per person, and the family as a whole as average per family member (se) at each two-week stage (* *p* < 0.05 Bonferroni corrected relative to baseline).

Table 2 shows six benefits for children remained at one year follow-up, far more than any other family member, indicating that the intervention had long-term benefits for children. Fruit and veg consumption increased by one portion/day during the intervention, which was maintained at follow up (0.75 portions/day) and one year later (see Figure 2).

#### 3.1.2. Female Parent—Specific Dietary Benefits

Table 2 shows reductions in sugars, fats, and total energy for the female parent relative to baseline during the intervention, and all of these remained (except total CHO) during the immediate follow-up. However, none of these persisted at one year follow-up. Consistent with children, reductions in sucrose were most prevalent.

#### 3.1.3. Male Parent—Specific Dietary Benefits

The intervention resulted in reduced sugar, fat, and energy intake for the male parent, with fat and energy remaining lower than baseline during the immediate follow-up (see Table 2 and Table 3). However, similarly to the female parent, these benefits were not evident one year later. Table 2 shows that the male parent was the least affected family member. Interestingly, Figure 2 shows that fruit and vegetable consumption remained elevated at one year follow-up, a benefit also observed for children at all time points.

### 3.2. Sugar Swaps during the Intervention (Swapping High Sugar Items for Low Sugar Items, Self-Reported)

As shown in Table 4, the female parent made the most swaps at 3.7 per day, with children making 3.5 swaps, and the male parent the fewest with 3 swaps. Most swaps were made for snacks and fewest for the evening meal as indicated by a main effect of Meal (F [3, 33] = 4.45, *p* < 0.05). No difference between swap type (food/drink) (*p* = 0.093) or between family members (*p* = 0.61) was observed. Children made significantly more drink swaps than food swaps (*p* < 0.05), as indicated by a Family Member * Type interaction (F [2, 22]3.75, *p* < 0.05).

## 4. Discussion

### 4.1. The Intervention Period

Significant benefits were achieved during the two-week intervention period when families were introduced to the campaign materials, with all members of the family reducing intake of sugar, fat, and energy relative to baseline. These benefits were sizeable; daily reductions of over 32 g sugar, 11 g fat, and 236 kcal for each family member, in combination with an additional portion of fruit and vegetables per day (in children only), would have significant benefits to long-term health if habitually maintained. The benefits during the intervention were most prevalent for children, with reduced sugar consumption most notable in snacks. These findings are perhaps not surprising given that the campaign was predominately targeted at children and the associated material largely focused on eating healthier snacks, by swapping high sugar items for low sugar alternatives. These findings are supported by previous research showing that public health interventions are moderately effective in the immediate period in which they are introduced. For example, a meta-analysis of interventions showed a pooled reduction of sugar sweetened beverage consumption by 76 mL/day [13]. During the intervention, families made on average 10 swaps per day (6 food and 4 drink), supporting previous research from the 2014 Smart Swaps campaign [10] where 74% of participants had made swaps relative to 30% for a control group. This suggests that the families were engaged with the materials and were able to understand the concept of the swaps.

### 4.2. Immediate Follow-Up

Encouragingly, 61% of the benefits achieved during the initial two weeks were maintained during the two-week follow-up when support ceased (although families had continued access to the materials they received at the outset). Specifically, during the two-week follow up, reductions in saturated fat were consistently maintained across all family members and there were also strong reductions in sucrose consumption for children (26 g day) and the female parent (14 g day), particularly from snacks. This shows that healthy snack eating behaviours adopted during the intervention persisted into the subsequent weeks for children and the female parent. Children may have been replacing high sugar/fat snacks with fruits/vegetables and low sugar alternatives, as supported by increased intake fruit and vegetable portions during follow-up.

### 4.3. One Year Follow-Up

For longer-term health gains, interventions must show changes in habitual behaviour. Perhaps the most striking finding from this research was that, for children, significant reductions in sugar, sucrose, fat, saturated fat, carbohydrate, and energy alongside increased fruit/veg intake were observed one year after the initial two-week intervention. It has been documented that 400,000 U.K. families signed up to a Change4Life dietary campaign in 2014 [10]; therefore, the dietary changes observed here could translate into significant population reductions in disease risk, including type 2 diabetes, CVD, dental problems, and obesity, as well as significant monetary savings for health services, as reported elsewhere in economic projection analysis [14,15]. This shows that meaningful long-term benefits can result from achievable daily changes to the diet.

### 4.4. Theoretical Considerations

Including children in the preparation and creation of meal plans has been reported to increase acceptance and reduce reluctance by all family members when attempting to introduce healthier eating habits [16], which could partially account for the relative success of this campaign. Other successful features of the Sugar Swaps materials may have been the emphasis on low-cost options and meal plans that are quick and easy to prepare; time constraints were highlighted as a key barrier to healthy eating in a European study of 5900 adults [17] and cost is one of the most frequently reported and well-established barriers to healthy eating across cultures [18,19,20,21].

However, it is important to recognise that the aim of this research was not to evaluate the elements of the Change4Life Sugar Swaps materials. Therefore, a limitation of this pilot study is that it is not possible to identify with any certainty which aspects of the campaign contributed to the successful outcomes reported here. Data were not collected that would allow an evaluation of which aspects of the promotional material were most effective, whether it be the meal plans, swaps suggestions, segregation of information by mealtimes, support from academic and professional bodies, or social aspects such as celebrity endorsement. A noteworthy characteristic is that all participants were members of Netmums.co.uk (who assisted in recruitment); therefore, it is possible that the sample may be more willing and likely to engage in online materials and associated health campaigns than the population average.

A strength of this research is the richness of the dataset; comprehensive food diaries were analysed for 49 participants over 8 weeks (a total of 2744 days). It is notable that average daily energy intake values for the parents across all stages of the study are lower than would be expected (1614 kcal and 1800 kcal for the female and male parents, respectively). In addition, energy intake was 215 kcal lower per child day at one year follow-up relative to baseline. Such a reduction is unlikely in growing children; therefore, under-reporting is a potential limitation here. However, the average calorie consumption per day reflects realistic quantities for children weighing on average 29 kg (1638 kcal per child). If systematic under reporting did occur, it is likely to have occurred across all arms of the study, and all analysis was relative to baseline. There is no evidence that under-reporting specifically increased at follow-up. For example, there were no changes for the three core macronutrients one year on relative to baseline for adults, which suggests consistency across time points. Of course, there are well established limitations of food diaries [22,23]. However satisfactory levels of agreement with plasma biomarkers have been reported in the Whitehall II cohort with written 7-day food diaries [24] and with 4-day online food diaries in older U.K. adults [25]. Online food diaries have also been reported to match energy expenditure assessed with indirect calorimetry [26]. It is important to acknowledge that the analysis of total sugar included naturally occurring sugars in fruits and vegetables, and it did not distinguish added or free sugar from naturally occurring sugar. The changes in fruit and vegetable intake thus undermine any potential interpretations relating to free or added sugars.

The absence of information on age and health measures is a limitation. Furthermore, the self-reported height/weight estimations were only taken at one year follow-up; therefore, assumptions cannot be made about the health status of the sample. All data were entered by one parent, which may introduce bias, and increases the likelihood of inaccurate reporting for other family members. This could account for the relatively small difference in average daily energy intake between the male and female parent (186 kcal). A further limitation is the relatively small sample size; however, this is offset by the richness of the data collected. For example, it is highly unusual to document comprehensive nutritional intake over a period of 56 days, especially in a whole family. The requirement of one member of the family to complete data entry for all members of the family likely explains the large amount of missing data and the high dropout rate. With this in mind, the findings should be interpreted with caution, as the data only represent families who were fully engaged with the procedures. Finally, the absence of a control group is a limitation as it is not possible to determine the degree to which the observed effects were influenced by simply enrolling in a study per se, or indeed any other external factors occurring over the duration of the study. Therefore, we cannot be certain that the observed benefits are directly caused by the campaign materials. Notwithstanding the limitations, this investigation resulted in the collection of a rich and detailed dataset, indicating that online diaries are an informative method of collecting dietary data over long periods.

## 5. Conclusions

The evidence shows that engaging in the Change4Life Sugar Swaps Campaign for a minimum of two weeks is associated with reduced consumption of sugar, fat, and energy in all members of the family. Importantly, these benefits extended beyond the first two weeks, with reduced intake of sugar and fat reported in the two weeks immediately following the intervention, and during a two-week period one year later. Increased intake of fruit and vegetables was also observed a year later. The greatest benefits were observed in children, who consumed far lower quantities of sugar and fat from snacks, and considering all nutrients, the largest reductions were clearly seen for sucrose consumption. In summary, this dataset shows that the Change4Life Sugar Swaps Campaign is an effective public health intervention for improving long-term dietary behaviour for the whole family.

## Figures and Tables

**Table 1:** Height and weight per family at one year follow-up (se; minimum–maximum).

	Female Parent (*n* = 14)	Male Parent (*n* = 12)	Average Child (*n* = 23 *)
Height (cm)	164.7 (1.6; 154–174)	179.1 (2.6; 163–191)	129.7 (4.2; 112–167)
Weight (kg)	64.2 (2.8; 48–88)	78.2 (2.9; 64–98)	28.7 (2.7; 19–56)
BMI (kg/m^2^) **	23.75	24.47	n/a

* Average number of children per family = 1.64; ** calculated as the average of all individual BMIs, not with height and weight from Table 1.

**Table 2:** Mean (se) daily intake per person (g) calculated from a maximum period of 14 days, fruit and veg = portion (80 g). * (*p* < 0.05) and ** (*p* < 0.01), significant difference from baseline following Bonferroni corrected post hoc tests. Mean (range) number of days data per family for each stage were 13.86 (12–14) for baseline, 13.93 (13–14) for intervention, 14 (14) for immediate follow-up, and 14 (14) for one year follow-up.

	Baseline	Intervention	Immediate Follow-Up	One Year Follow-Up	Main Effect of TIME (df 339)	Time * Meal Interaction (df 9117)
Child (*n* = 23)						
Energy (kcal)	1792 (90)	1555 * (42)	1630 (66)	1576 * (59)	0.001	0.068
Carbohydrate	251 (15)	215 (7)	221 (11)	213 * (10)	0.002	0.088
Fibre	17 (1)	20 * (1)	19 (1)	18 (1)	0.001	0.077
Fructose	19 (2)	19 (2)	18 (2)	19 (3)	0.956	0.704
Glucose	19 (3)	16 (2)	16 (2)	16 (2)	0.429	0.591
Lactose	14 (2)	14 (1)	14 (2)	15 (2)	0.982	0.007
Sucrose	46 (4)	21 ** (2)	26 ** (3)	28 ** (3)	<0.000001	<0.000001
Sugar	115 (12)	78 ** (5)	84 * (7)	89 (8)	0.0002	0.011
Fat	66 (3)	54 ** (2)	59 (3)	57 * (3)	0.0001	0.012
Sat Fat	25 (1)	19 ** (1)	21 (1)	21 * (1)	0.0002	0.0002
Protein	64 (4)	66 (3)	65 (3)	66 (2)	0.799	0.255
Salt	4.8 (0.3)	4.4 (0.2)	4.8 (0.3)	4.6 (0.2)	0.083	0.91
Vitamin C (mg)	59 (16)	65 (16)	84 (8)	90 (28)	0.356	0.028
Fruit and veg	2.4 (0.3)	3.4 ** (0.3)	3.2 * (0.4)	3.5 ** (0.2)	0.0001	n/a
Energy (kcal)	1762 (79)	1473 ** (79)	1530 * (81)	1693 (96)	0.012	0.018
Carbohydrate	226 (12)	189 * (12)	198 (13)	201 (15)	0.014	0.04
Fibre	18 (1)	20 (2)	19 (2)	19 (2)	0.481	0.614
Fructose	17 (2)	15 (2)	17 (3)	17 (2)	0.658	0.026
Glucose	16 (2)	13 (2)	16 (2)	14 (2)	0.236	0.051
Lactose	14 (2)	13 (2)	12 (2)	12 (2)	0.293	0.006
Sucrose	36 (3.9)	15 ** (1.5)	21* (3.2)	26 (3.5)	0.00002	0.0003
Sugar	92 (7)	60 ** (5)	74 * (7)	76 (8)	0.003	0.052
Fat	67 (4)	53 ** (3)	56 ** (4)	68 (5)	0.003	0.168
Sat Fat	24 (2)	17 ** (2)	19 ** (2)	24 (2)	0.006	0.41
Protein	68 (3)	68 (4)	67 (4)	80 (10)	0.372	0.4
Salt	4.7 (0.3)	4.2 (0.3)	4.5 (0.4)	4.8 (0.3)	0.063	0.026
Vitamin C (mg)	68 (15)	71 (13)	73 (14)	96 (21)	0.365	0.919
Fruit and veg	2.3 (0.3)	2.9 (0.4)	3.1 (0.5)	2.9 (0.4)	0.15	n/a
Male parent (*n* = 12)						
Energy (kcal)	1959 (99)	1636 * (70)	1731 * (81)	1873 (95)	0.00006	0.187
Carbohydrate	237 (16)	198 * (13)	213 (16)	227 (17)	0.002	0.084
Fibre	19 (1)	20 (2)	19 (1)	20 (1)	0.456	0.075
Fructose	13 (2)	10 (2)	12 (2)	14 (2)	0.055	0.292
Glucose	13 (2)	9 (1)	11 (2)	13 (2)	0.036	0.309
Lactose	14 (2)	5 (1)	8 (2)	14 (2)	0.45	0.733
Sucrose	37 (5)	15 ** (3)	23 (4)	33 (7)	0.0002	0.008
Sugar	85 (9)	52 ** (6)	65 (9)	78 (11)	0.0004	0.457
Fat	77 (3)	62 ** (3)	67 * (2)	73 (5)	0.003	0.42
Sat Fat	27 (2)	21 (2)	22 * (1)	23 (2)	0.006	0.2
Protein	79 (6)	75 (4)	73 (3)	78 (4)	0.406	0.602
Salt	6 (0.4)	5.3 (0.3)	5.6 (0.4)	5.5 (0.3)	0.111	0.308
Vitamin C (mg)	49 (9)	46 (7)	45 (7)	69 (11)	0.04	0.417
Fruit and veg	1.5 (0.2)	1.9 (0.3)	1.8 (0.2)	2.4 * (0.3)	0.004	n/a

**Table 3:** Significant reductions only in intake per day relative to baseline for each member of the family (Bonferroni corrected *p* < 0.05) (breakfast = observed change for breakfast, snack = change observed for snack, lunch = observed change for lunch, EM = change observed for evening meal). One portion of fruit and vegetables = 80 g. Mean (range) number of days data per family for each stage were 13.86 (12–14) for baseline, 13.93 (13–14) for intervention, 14 (14) for immediate follow-up, and 14 (14) for one year follow-up. * represents an increase.

	Intervention	Immediate Follow-Up	One Year Follow-Up
Child (*n* = 23)			
Energy	237 kcal	-	215 kcal
Carbohydrate	-	-	38.6 g
Fibre *	3.1 g	-	-
Sugar (s)	36.8 g Breakfast 7.3 g EM 7 g Sucrose 24.4 g Sucrose breakfast 4.7 g Sucrose EM 4.4 g Sucrose snacks 14.1 g	31.2 g Sucrose 25.7 g Sucrose snacks 12.6 g	EM 6.1 g Sucrose 17.9 g Sucrose snacks 11 g
Fat	11.7 g Snack 6.5 g	-	8.6 g Breakfast 2.5 g
Sat Fat	5.8 g Snack 3.3 g	Snack 1.9 g	3.8 g
Fruit and Veg *	0.96 portions	0.75 portions	1.03 portions
Female parent (*n* = 14)			
Energy	289 kcal EM 119 kcal	232 kcal Lunch 86 kcal	-
Carbohydrate	36.5 g EM 16.6 g	-	-
Sugar (s)	32.1 g Sucrose 20.7 g Sucrose lunch 3.8 g Sucrose EM 5 g Sucrose snacks 5.3 g	Sucrose 14.3 g Sucrose snacks 4 g	-
Fat	14.5 g	10.8 g	-
Saturated Fat	7 g	5.8 g	-
Male parent (*n* = 12)			
Energy	323 kcal	228 kcal	-
Carbohydrate	38.6 g	-	-
Sugar (s)	32.9 g Sucrose 21.9 g Sucrose breakfast 3.3 g Sucrose snacks 11 g	-	-
Fat	14.8 g	10.4 g	-
Saturated Fat	-	5.2 g	-
Fruit and Veg *	-	-	0.85 portions

**Table 4:** Average swaps over 14 days for each family member separated by type (food or drink) and meal (breakfast, lunch, evening meal, and snacks). Data are average per a single child where a family has more than one child. Mean (range) number of days data per family for each stage were 13.86 (12–14) for baseline, 13.93 (13–14) for intervention, 14 (14) for immediate follow-up, and 14 (14) for one year follow-up.

	Female Parent (*n* = 14)			Male Parent (*n* = 12)			Average Child (*n* = 23)			Family
	Food	Drink	Total	Food	Drink	Total	Food	Drink	Total	Total
Breakfast	7.7 (1.9)	7.2 (1.8)	14.9	6.8 (1.3)	7.3 (2.2)	14.1	8 (1.1)	4.6 (1.2)	12.6	41.6
Lunch	4.9 (1.2)	6.4 (1.5)	11.1	5.7 (2)	4.4 (1.4)	10.1	5 (1.1)	4.7 (1.3)	9.7	30.9
Evening Meal	4 (0.8)	4.6 (1.7)	8.6	3.5 (0.9)	3.4 (1.5)	6.9	4.9 (1.1)	3.3 (1.1)	8.2	23.7
Snacks	10.6 (2)	6.1 (2.5)	16.7	6.4 (1.9)	4.8 (1.7)	11.2	13.2 (1.8)	5 (1.6)	18.2	46.1
Total	27.3 (3.3)	24.3 (6.3)	51.6	22.3 (5)	19.8 (5.7)	42.1	31.1 (3.1)	17.6 (4.2)	48.7	142.4
Mean/day	1.9	1.7	3.7	1.6	1.4	3	2.2	1.3	3.5	10.2

## Data Availability

Data can be made available by request to Daniel Lamport by email: daniel.lamport@reading.ac.uk.
